# Prognostic effect of radiotherapy in breast cancer patients underwent immediate reconstruction after mastectomy

**DOI:** 10.3389/fonc.2022.1010088

**Published:** 2022-11-02

**Authors:** Luyao Dai, Hanxiao Cui, Yuanhang Bao, Liqun Hu, Zhangjian Zhou, Shuai Lin, Xin Zhang, Hao Wu, Huafeng Kang, Xiaobin Ma

**Affiliations:** ^1^ Department of Oncology, The Second Affiliated Hospital of Xi’an Jiaotong University, Xi’an, Shaanxi, China; ^2^ School of Basic Medical Sciences, Xi’an Key Laboratory of Immune Related Diseases, Xi’an Jiaotong University, Xi’an, Shaanxi, China

**Keywords:** radiotherapy, breast cancer, immediate breast reconstruction (IBR), the surveillance, epidemiology, and end results (SEER), nomograms

## Abstract

**Introduction:**

It is still unclear whether radiotherapy affects the long-term survival of breast cancer (BC) patients after immediate breast reconstruction (IBR). This study aims to evaluate the actual prognostic impact of radiotherapy on BC patients undergoing IBR, and to construct survival prediction models to predict the survival benefit of radiotherapy.

**Methods:**

Data on eligible BC patients were retrieved from the Surveillance, Epidemiology, and End Results (SEER) database. Competing risk models were used to assess breast cause-specific death (BCSD) and non-breast cancer cause-specific death (NBCSD). Kaplan‐Meier curve, Cox risk regression model and forest map were used to evaluate and demonstrate overall survival (OS) and breast cancer-specific survival (BCSS). Survival prediction nomograms were used to predict OS and BCSS probabilities.

**Results:**

A total of 22,218 patients were selected, 24.9% received radiotherapy and 75.1% were without radiotherapy. Competing risk models showed that whether BCSD or NBCSD, the cumulative long-term risk of death in the radiotherapy group was higher than that in the non-radiotherapy group. The Kaplan‐Meier curve showed that patients with different lymph node metastasis had different radiotherapy benefits. Multivariate stratified analysis showed that radiotherapy after autologous reconstruction was associated with poor BCSS in patients with stage N0, and radiotherapy after autologous reconstruction and combined reconstruction improved OS and BCSS in patients with stage N3. The C-indexes of nomogram (between 0.778 and 0.847) and calibration curves showed the good prediction ability of survival prediction model.

**Conclusions:**

Radiotherapy can improve OS and BCSS in N3 stage BC patients undergoing immediate autologous reconstruction after mastectomy. The practical nomograms can be used to predict OS and BCSS of patients with or without radiotherapy, which is helpful for individualized treatment.

## Introduction

Breast cancer (BC) is the most common cancer in women (1). In 2020, BC has surpassed lung cancer as the leading cause of global cancer incidence. It is also the fifth leading cause of cancer mortality worldwide, and one of the highest burden of cancers in the world (2, 3). Mastectomy is one of the traditional and preferred surgical treatment methods (4–6). However, partial or complete mastectomy can alter the patients’ body shape, and have adverse social, sexual or psychological consequences. Patients who undergo breast reconstruction after mastectomy have been reported to have a better quality of life (7–9). As a result, breast reconstruction has become increasingly popular in recent years, generally including immediate breast reconstruction (IBR) and delayed breast reconstruction (DBR). Some studies have suggested that IBR is superior to DBR due to lower cost and surgical risk, and higher patient satisfaction (10–12). Currently, there is no evidence that IBR increases the risk of postoperative recurrence and death (13).

Postmastectomy radiotherapy (PMRT), as an effective postoperative adjuvant treatment to prevent recurrence and improve survival, is being actively implemented (14–16). Evidence indicates that patients receiving PMRT have significantly improved survival and a reduced risk of local recurrence (17, 18). According to the authoritative guidelines issued by the American Society of Clinical Oncology (ASCO) in 2001, the primary indication of PMRT is tumors larger than 5 cm or more than 3 positive axillary lymph nodes (19). In the following 10 years, the National Comprehensive Cancer Network (NCCN) has expanded the indications of PMRT. PMRT is “considered” and “strongly considered” for patients with tumors ≤ 5cm and 1-3 positive lymph nodes, respectively (20). Nevertheless, for patients requiring PMRT, breast reconstruction is often delayed due to adverse tissue changes associated with radiotherapy (21–23). Possible side effects include capsular contracture, tissue fibrosis and edema, indicating a higher risk of infection (24, 25). Münire Kayahan et al. (26) reported that patients who received radiotherapy after reconstruction were more frequently found to suffer complications and implant failure. Christante et al. (27) showed that more than 30% of patients who received radiotherapy after IBR required the removal of implants. Despite this, the number of patients undergoing radiotherapy after IBR continues to rise (20). The necessity and usefulness of PMRT are complicated for patients with breast reconstruction (28). Although a few studies have shown that IBR does not affect the implementation of radiotherapy, most studies investigating the role of radiotherapy in patients with IBR focused on cosmesis effects, rather than survival outcomes (29–31).

The current national reconstruction practice, particularly in the patients who are more challenging to undergoing PMRT, is less well known (32). The purpose of this study is to investigate the prognostic effects of radiotherapy on breast cancer patients undergoing immediate reconstruction after mastectomy, and to evaluate the potential survival benefits individually.

## Methods

### Data source and study population

The data for this study were obtained from the Surveillance, Epidemiology, and End Results (SEER) database. Based on the software SEER*Stat version 8.3.9.1, we extracted the required information (with additional treatment fields) from the SEER database for 18 cancer registries from 1975 to 2016. The radiotherapy data were obtained from a separate application. As one of the most representative large cancer databases in the United States, data from the SEER database are publicly available. Informed consent is not required because there is no private information involved. The Ethics Committee of the Second Affiliated Hospital of Xi’an Jiaotong University approved this study.

The analysis included women with microscopically confirmed breast cancer who underwent immediate mastectomy reconstruction between 2010 and 2015. Exclusion criteria are as follows: 1) more than one primary tumor; 2) details were unknown or unclear; 3) diagnosis only be made by autopsy or death certificate; 4) survival time was equal to zero. Patients in SEER were followed up to death, and any patient who died after the follow-up deadline was recoded as alive patients after the deadline. Through screening, 22,218 eligible patients were enrolled in this study. Individual data for each case included age, race, marital status, histological type, grade, tumor-node-metastasis (TNM) stage, breast cancer subtype, reconstruction procedure, and whether to have radiotherapy. Marital status was regrouped into two groups: Married group and not married group. Single (never married), separated, divorced, widowed or domestic partner were grouped into the not married group. Histological types were classified according to the International Classification of Diseases in Oncology, Third Edition (ICD‐O‐3) into 4 categories, ductal (8500), ductal/lobular (8520), lobular (8522) and other. TNM stage was based on the seventh edition of the American Joint Council on Cancer (AJCC). Additionally, the SEER database reports breast reconstruction methods within 4 months after primary mastectomy, including autologous reconstruction, implant reconstruction, and combined reconstruction.

### Statistical analysis

Age was a continuous variable. So in order to select the best cut point, we stratified the age of the patients with the X-tile software. Descriptive statistical analysis of patient distribution was performed using frequency and proportion. The Chi-square test was used to compare the clinical distribution characteristics of patients with different radiotherapy conditions. The co-primary endpoints of this study were overall survival (OS) and breast cancer-specific survival (BCSS). Causes of death in breast cancer patients can be divided into breast cancer-specific death (BCSD) and non-breast cancer-specific death (NBCSD). To preliminarily describe the risks under different radiotherapy conditions, cumulative incidence maps were constructed by using competitive risk models. The population was further stratified according to the preliminary analysis results, and the Kaplan-Meier curves and log-rank tests were used to assess the impact of different radiotherapy conditions on patients’ prognosis. The Cox proportional risk model was used to calculate the hazard ratio (HR) and its corresponding 95% confidence interval (CI). And the visual results of multivariate analysis were presented in the form of forest map. Subsequently, to further evaluate the impact of radiotherapy on different reconstruction methods, we calculated the adjusted hazard ratio (AHR) and the corresponding 95% CI between patients receiving radiotherapy and patients not receiving radiotherapy, and stratified by cancer stage to deal with potential bias. Finally, we established nomograms to predict patients’ survival, and evaluated the prediction accuracy with concordance indexes (C-indexes) and calibration curves.

All statistical analyses were completed by R software (Version 4.0.3; http://www.r-project.org) and related R packages, mainly including “survival”, “cmprsk”, “rms”, “ggplot2” packages and so on. A two-sided P value less than 0.05 was determined to be statistically significant.

## Results

### Patients’ characteristics and radiotherapy trends

**Figure 1:**
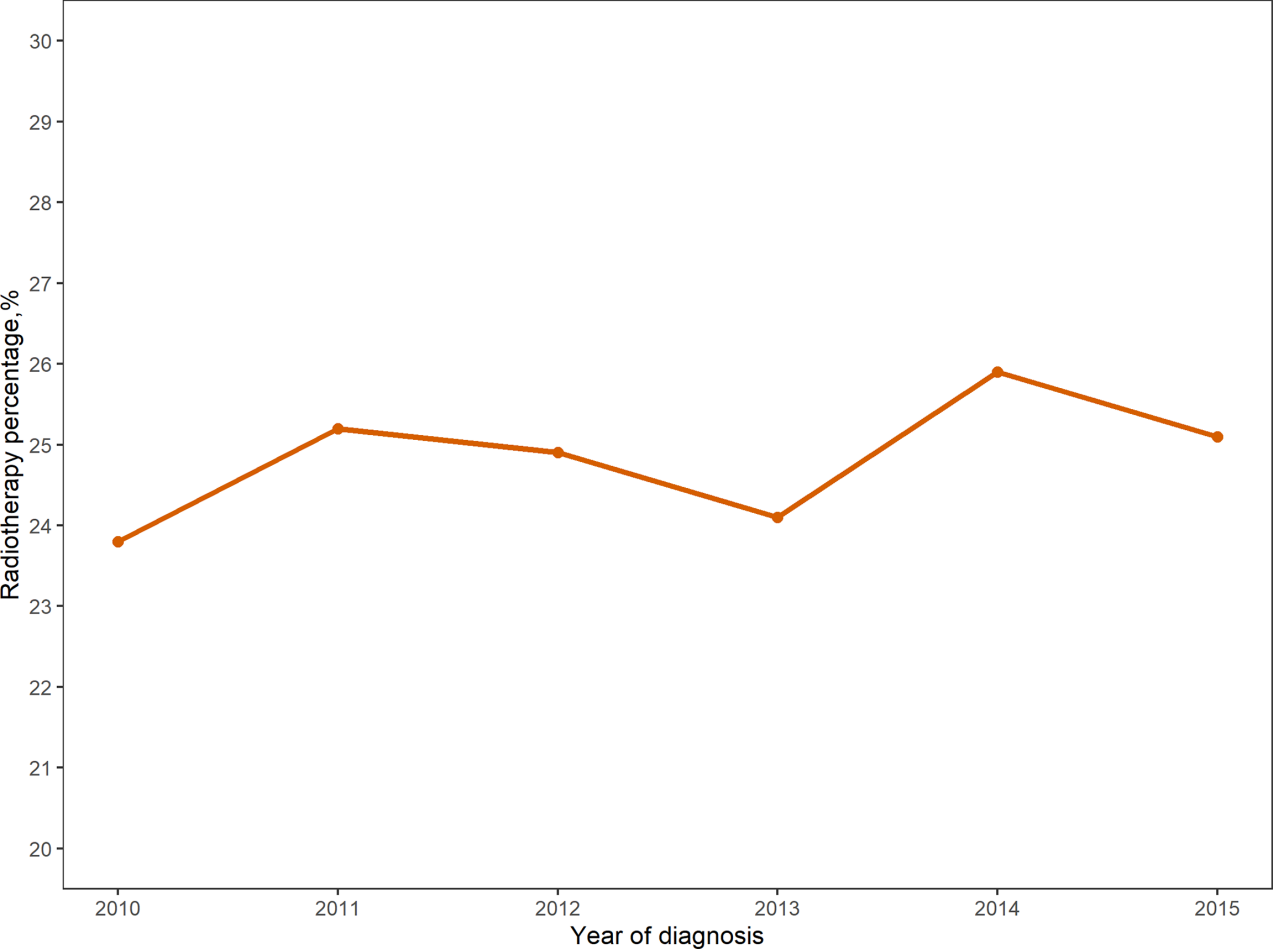
Proportion of patients undergoing immediate breast reconstruction after mastectomy receiving radiotherapy from 2010-2015.

**Figure 2:**
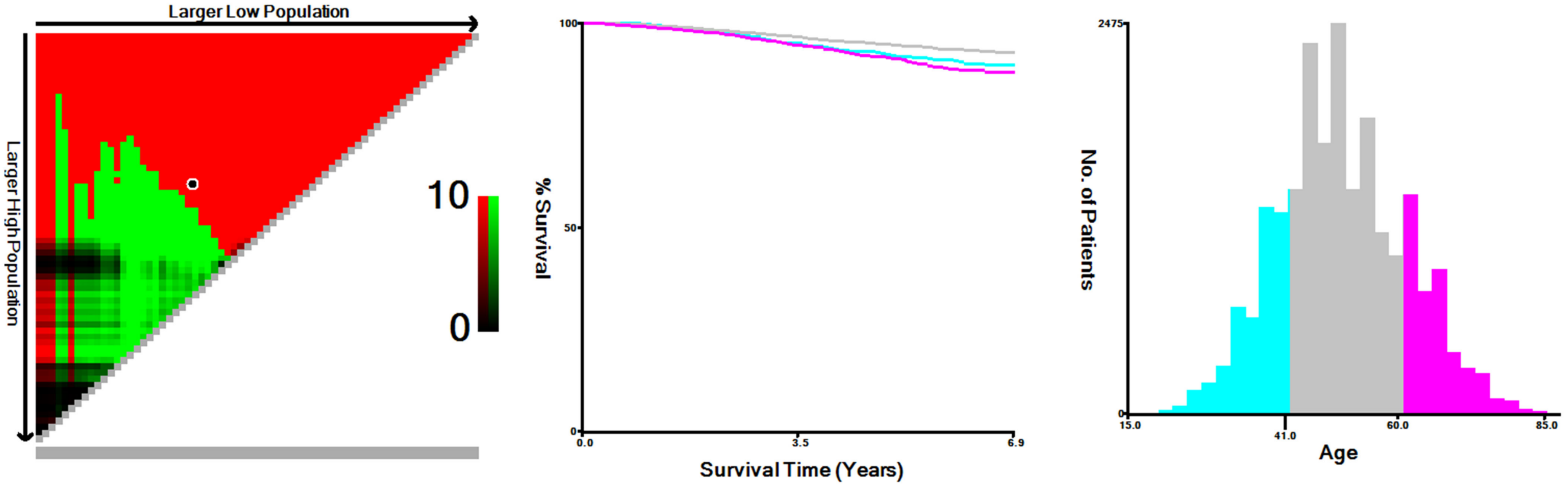
Age of patients divided by X-tile software at the best cut point.

From 2010 to 2015, the analysis included 22,218 female patients with breast cancer who underwent IBR after mastectomy. Among them, 5,529 patients received PMRT and 16,689 were without PMRT. The overall percentage of patients receiving PMRT did not change much over the years, but there was still a slight upward trend (**Figure 1**). As shown in **Table 1**, significant distribution differences in age, race, histological type, grade, TNM stage, and breast cancer subtype were observed between the radiotherapy and non-radiotherapy subgroups. Based on Kaplan-Meier method, we used X-tile program to determine the optimal cut-off points of age as 40 and 60 years old (**Figure 2**). Therefore, age was divided into three groups: < 40 years old, 40-60 years old and > 60 years old. Patients in both the radiotherapy and non-radiotherapy groups were predominantly 40-60 years old, white, married, histological type of ductal carcinoma, had grade II and III tumors, no distant metastasis, breast cancer subtype of HR+/HER2-, and underwent implant reconstruction. The difference was that T stage was mostly T2 (45.2%) and N stage was mostly N1 (52.9%) in the radiotherapy group, while T1 (60.4%) and N0 (72.6%) accounted for most in the non-radiotherapy group.

**Table 1:** Baseline demographic characteristics of patients who were reconstructed (n=22218).

	Radiotherapy	Non-radiotherapy	
variable	N=5529(%)	N=16689(%)	P
Age, y			<0.001
<40	1174 (21.2)	2125 (12.7)	
40-60	3481 (63.0)	11198 (67.1)	
>60	874 (15.8)	3366 (20.2)	
Race			<0.001
White	4404 (79.7)	13741 (82.3)	
Black	712 (12.9)	1533 (9.2)	
Other^a^	413 (7.5)	1415 (8.5)	
Marital_status			0.074
Married	3787 (68.5)	11646 (69.8)	
Not married^b^	1742 (31.5)	5043 (30.2)	
Histologic_type			<0.001
Ductal	4042 (73.1)	12548 (75.2)	
Ductal/lobular	394 (7.1)	1104 (6.6)	
Lobular	713 (12.9)	1580 (9.5)	
Other	380 (6.9)	1457 (8.7)	
Grade			<0.001
I	529 (9.6)	3364 (20.2)	
II	2476 (44.8)	7513 (45.0)	
III	2512 (45.4)	5759 (34.5)	
IV	12 (0.2)	53 (0.3)	
T			<0.001
T1	1370 (24.8)	10083(60.4)	
T2	2500 (45.2)	5687 (34.1)	
T3	1389 (25.1)	759 (4.5)	
T4	270 (4.9)	160 (1.0)	
N			<0.001
N0	985 (17.8)	12108 (72.6)	
N1	2924 (52.9)	3838 (23.0)	
N2	1070 (19.4)	498 (3.0)	
N3	550 (9.9)	245 (1.5)	
M			<0.001
M0	5404 (97.7)	16544 (99.1)	
M1	125 (2.3)	145 (0.9)	
Subtype			<0.001
HR+/HER2+	878 (15.9)	2203 (13.2)	
HR+/HER2-	3639 (65.8)	11710 (70.2)	
HR-/HER2+	359 (6.5)	925 (5.5)	
Triple negative	653 (11.8)	1851 (11.1)	
Reconstruction			0.421
Autologous	1927 (34.9)	5970(35.8)	
Implant	2706 (48.9)	8013 (48.0)	
Combined	896 (16.2)	2706 (16.2)	

^a^Other: American Indian/AK Native, Asian/Pacific Islander.

^b^Not married: single (never married), separated, divorced, widowed, or domestic partner.

### Cumulative incidence of death and competing risk analysis

**Figure 3:**
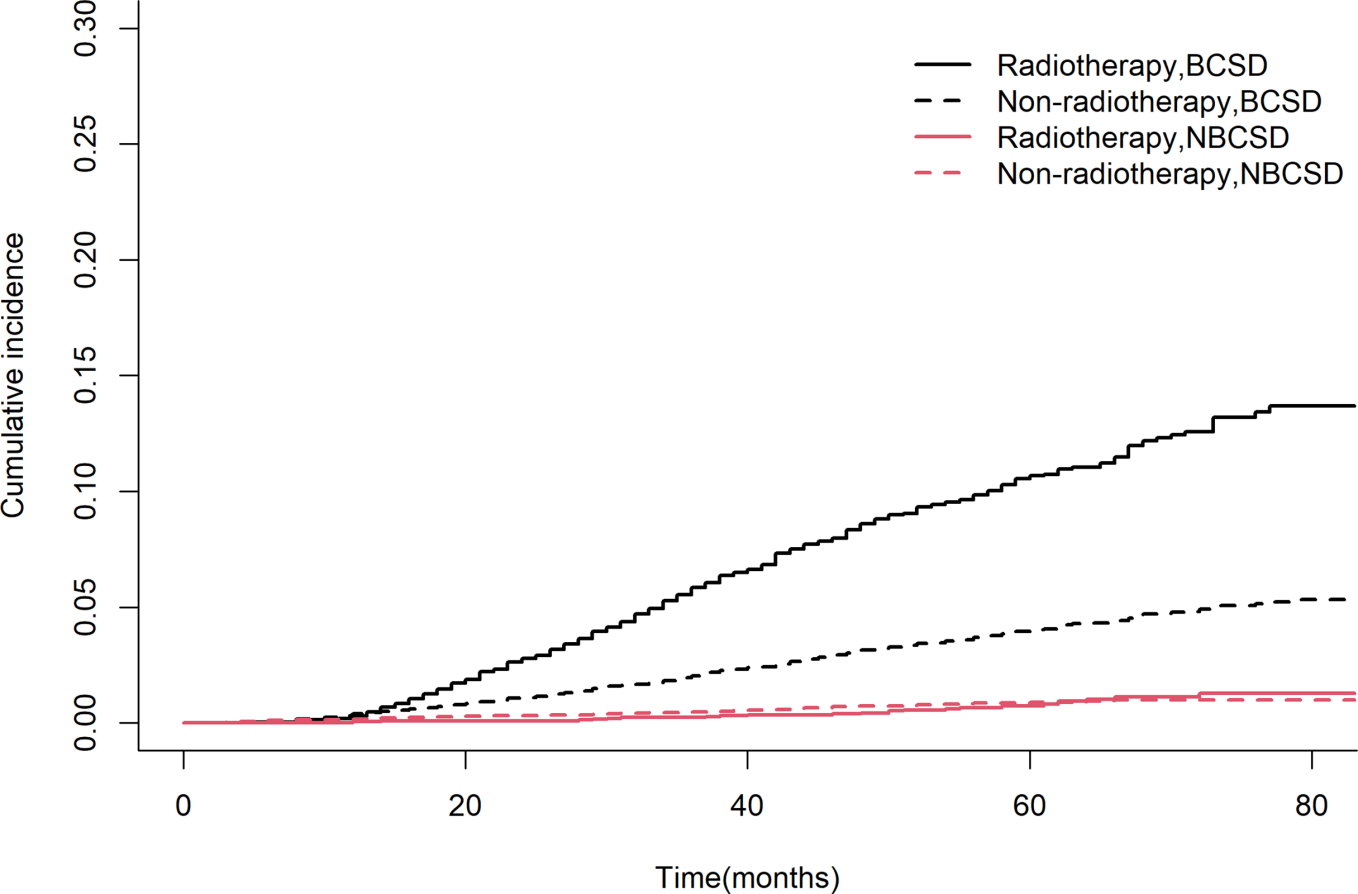
Competing risk models. BCSD, breast cancer-specific death; NBCSD, non-breast cancer-specific death.

A total of 976 patients (4.4%) died, of which 846 (86.7%) died from BC and 130 (13.3%) died from non-BC. **Table 2** showed the cumulative incidence of BCSD and NBCSD at 1-, 3-, and 5- year. The cumulative incidence of BCSD in the radiotherapy group was higher than that in the non-radiotherapy group after 13 months (**Figure 3**). However, the cumulative incidence rate of NBCSD was lower in the two groups, and slightly higher in the radiotherapy group than in the non-radiotherapy group after 62 months. After controlling competing risk events, there was a statistically significant difference in the cumulative risk of BCSD between the two groups (P < 0.0001).

**Table 2:** The 1-, 3-, and 5-year cumulative incidence of BCSD^a^ and NBCSD^b^.

	Radiotherapy (%)	Non-radiotherapy (%)
BCSD		
1-Year CIF^c^	0.29	0.39
3-Year CIF	5.84	2.04
5-Year CIF	10.67	4.02
NBCSD		
1-Year CIF	0.05	0.16
3-Year CIF	0.25	0.48
5-Year CIF	0.73	0.88

^a^BCSD; breast cancer-specific death, ^b^NBCSD; non-breast cancer-specific death, ^c^CIF; cumulative incidence function.

### OS and BCSS outcomes

**Figure 4:**
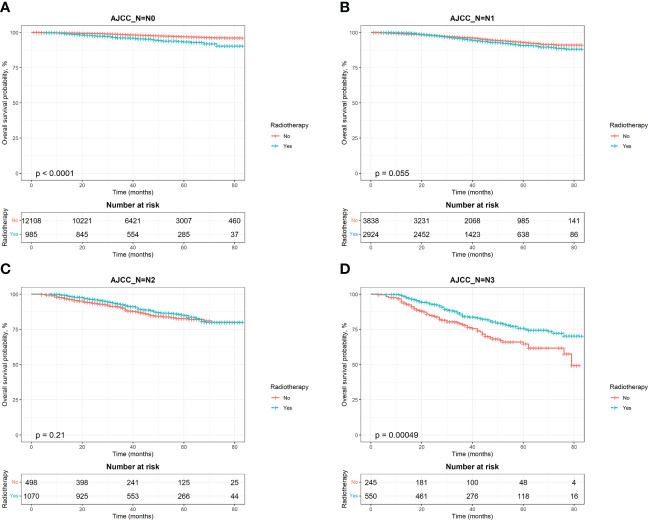
Kaplan–Meier survival curves of overall survival after stratifying by AJCC N stage. **(A)**, AJCC_N=N0; **(B)**, AJCC_N=N1; **(C)**, AJCC_N=N2; **(D)**, AJCC_N=N3.

**Figure 5:**
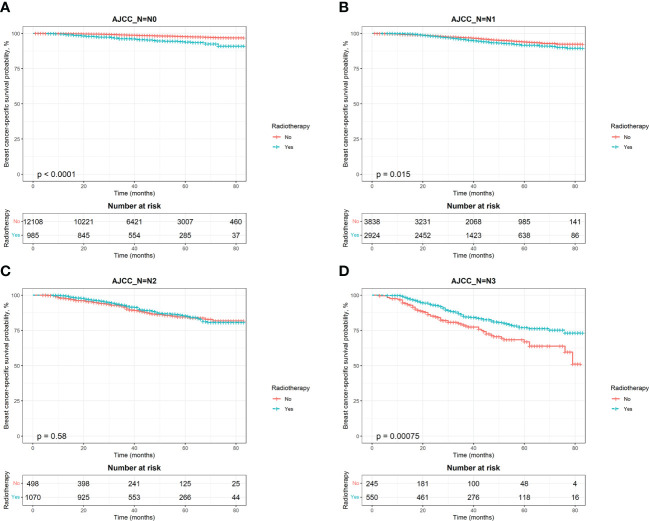
Kaplan–Meier survival curves of breast cancer-specific survival after stratifying by AJCC N stage. **(A)**, AJCC_N=N0; **(B)**, AJCC_N=N1; **(C)**, AJCC_N=N2; **(D)**, AJCC_N=N3.

To estimate OS and BCSS in breast cancer patients, we stratified the population by AJCC N stage and generated Kaplan‐Meier curves based on whether patients received PMRT. The results showed that patients with different N stages had different radiotherapy benefits. The OS of patients with stage N0 was worse after radiotherapy, while that of patients with stage N3 was better after radiotherapy (**Figures 4A, D**). Further log‐rank tests confirmed that the difference was statistically significant (P < 0.0001). The effect of PMRT on OS in N1 and N2 patients (**Figures 4B, C**) was not significant (P > 0.05). As for BCSS, it was worse in N1 patients after radiotherapy (P = 0.015), and the results of other 3 stages’ patients were consistent with OS (**Figure 5**). Median follow-up time for both OS and BCSS was 42 months (95% CI, 42-43 months).

**Figure 6:**
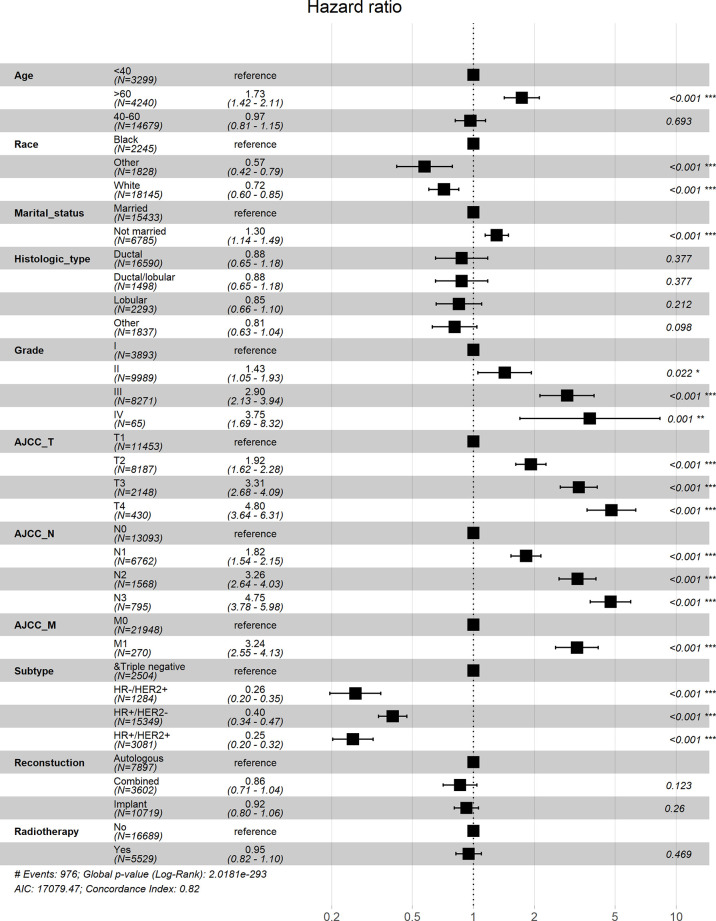
Forest map visualizing Cox multivariate regression of overall survival of patients. *: <0.05; **: <0.01; ***: <0.001.

**Figure 7:**
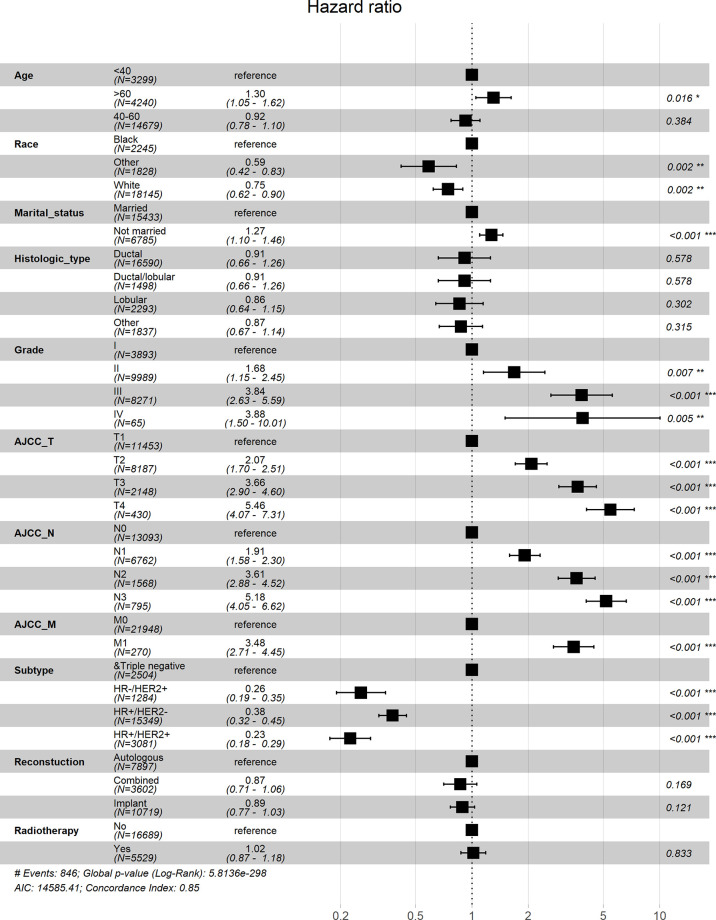
Forest map visualizing Cox multivariate regression of breast cancer-specific survival of patients. *: <0.05; **: <0.01; ***: <0.001.

Univariate Cox analysis showed that in both OS and BCSS, there were significant differences in age, race, marital status, histological type, grade, TNM stage, subtype, reconstruction method, and radiotherapy condition among all subgroups. Considering that the correlation among characteristics may lead to bias, we further conducted a multivariable Cox analysis (**Table 3**). The results showed that histological type, reconstruction method and radiotherapy condition were no longer statistically correlated with OS and BCSS (P > 0.05). Compared with patients < 40 years old, patients over 60 years old had worse OS (HR, 1.733; 95% CI, 1.421-2.113; P < 0.001) and BCSS (HR, 1.304; 95% CI, 1.050-1.619; P = 0.016). As for race subgroups, the prognosis of patients with white and other races were better than blacks. Those who were not married had poorer OS (HR, 1.304; 95% CI, 1.143-1.487; P < 0.001) and BCSS (HR, 1.269; 95% CI, 1.102-1.463; P < 0.001) than the married group. As expected, patients’ outcomes were inversely proportional to tumor grade, size, and number of lymph nodes. Patients with distant metastasis of tumor lesions also have worse OS (HR, 3.244; 95% CI, 2.547-4.131; P < 0.001) and BCSS (HR, 3.477; 95% CI, 2.715-4.454; P < 0.001). The results were also illustrated by the form of forest maps (**Figures 6** and **7**).

**Table 3:** OS and BCSS in univariate and multivariate analyses.

	OS				BCSS			
	Univariate		Multivariate		Univariate		Multivariate	
Variable	HR (95%CI)	P	HR (95%CI)	P	HR (95%CI)	P	HR (95%CI)	P
Age, y								
<40	Reference		Reference		Reference		Reference	
40-60	0.697 (0.588-0.826)	<0.001	0.966 (0.813-1.147)	0.693	0.649 (0.546-0.773)	<0.001	0.925(0.775-1.103)	0.384
>60	1.124 (0.926-1.363)	0.237	1.733 (1.421-2.113)	<0.001	0.817 (0.662-1.009)	0.060	1.304 (1.050-1.619)	0.016
Race								
Black	Reference		Reference		Reference		Reference	
White	0.485 (0.412-0.570)	<0.001	0.716 (0.604-0.848)	<0.001	0.481 (0.404-0.573)	<0.001	0.747 (0.623-0.896)	0.002
Other	0.343 (0.252-0.469)	<0.001	0.574 (0.419-0.788)	<0.001	0.340 (0.243-0.475)	<0.001	0.589 (0.419-0.827)	0.002
Marital status								
Married	Reference		Reference		Reference		Reference	
Not married	1.636 (1.44-1.858)	<0.001	1.304 (1.143-1.487)	<0.001	1.593 (1.388-1.827)	<0.001	1.269 (1.102-1.463)	<0.001
Histologic type								
Ductal	Reference		Reference		Reference		Reference	
Ductal/lobular	0.693 (0.519-0.924)	0.013	0.875 (0.651-1.176)	0.377	0.681 (0.499-0.930)	0.016	0.913 (0.664-1.257)	0.578
Lobular	0.686 (0.540-0.870)	0.002	0.849 (0.656-1.098)	0.212	0.617 (0.472-0.807)	<0.001	0.859 (0.643-1.147)	0.302
Other	0.778 (0.606-0.998)	0.048	0.808 (0.629-1.040)	0.098	0.813 (0.626-1.056)	0.121	0.873 (0.671-1.138)	0.315
Grade								
I	Reference		Reference		Reference		Reference	
II	2.079 (1.540-2.806)	<0.001	1.428 (1.054-1.935)	0.022	2.596 (1.789-3.768)	<0.001	1.680(1.153-2.448)	0.007
III	6.287 (4.728-8.360)	<0.001	2.899 (2.133-3.940)	<0.001	9.230 (6.468-13.171)	<0.001	3.836 (2.632-5.590)	<0.001
IV	6.794 (3.083-14.972)	<0.001	3.753 (1.693-8.320)	0.001	7.727 (3.011-19.831)	<0.001	3.879 (1.503-10.014)	0.005
T								
T1	Reference		Reference		Reference		Reference	
T2	3.210 (2.723-3.784)	<0.001	1.921 (1.616-2.285)	<0.001	3.781 (3.140-4.554)	<0.001	2.070 (1.704-2.515)	<0.001
T3	6.172 (5.100-7.468)	<0.001	3.309 (2.680-4.087)	<0.001	7.638 (6.189-9.426)	<0.001	3.656 (2.904-4.604)	<0.001
T4	14.452 (11.289-18.501)	<0.001	4.797 (3.643-6.315)	<0.001	18.372 (14.106-23.929)	<0.001	5.455 (4.071-7.311)	<0.001
N								
N0	Reference		Reference		Reference		Reference	
N1	2.394 (2.048-2.798)	<0.001	1.819 (1.535-2.154)	<0.001	2.688 (2.263-3.194)	<0.001	1.910 (1.585-2.302)	<0.001
N2	5.207 (4.319-6.279)	<0.001	3.261 (2.642-3.025)	<0.001	6.237 (5.102-7.625)	<0.001	3.606 (2.877-4.519)	<0.001
N3	9.966 (8.219-12.085)	<0.001	4.751 (3.777-5.978)	<0.001	12.014 (9.775-14.765)	<0.001	5.183 (4.055-6.625)	<0.001
M								
M0	Reference		Reference		Reference		Reference	
M1	10.050 (8.072-12.51)	<0.001	3.244 (2.547-4.131)	<0.001	11.370 (9.083-14.230)	<0.001	3.477 (2.715-4.454)	<0.001
Subtype								
Triple negative	Reference		Reference		Reference		Reference	
HR+/HER2+	0.251 (0.200-0.315)	<0.001	0.255 (0.202-0.320)	<0.001	0.227 (0.178-0.289)	<0.001	0.225 (0.176-0.289)	<0.001
HR+/HER2-	2.254 (0.220-0.292)	<0.001	0.400 (0.341-0.470)	<0.001	0.226 (0.194-0.262)	<0.001	0.378 (0.319-0.448)	<0.001
HR-/HER2+	0.347 (0.262-0.460)	<0.001	0.262 (0.196-0.350)	<0.001	0.347 (0.259-0.465)	<0.001	0.257 (0.190-0.346)	<0.001
Reconstruction								
Autologous	Reference		Reference		Reference		Reference	
Implant	0.833 (0.727-0.954)	0.008	0.924 (0.805-1.060)	0.260	0.799 (0.690-0.924)	0.003	0.889 (0.767-1.032)	0.121
Combined	0.788 (0.651-0.953)	0.014	0.860 (0.710-1.042)	0.123	0.787 (0.643-0.964)	0.021	0.867 (0.707-1.063)	0.169
Radiotherapy								
No	Reference		Reference		Reference		Reference	
Yes	2.363 (2.082-2.682)	<0.001	0.948 (0.820-1.095)	0.469	2.708 (2.366-3.100)	<0.001	1.017 (0.872-1.185)	0.833

### Subgroup analysis stratified by AJCC N stage

To solve the potential deviation of patients with different lymph node metastasis, we stratified the population according to AJCC N stage, and calculated the AHRs of radiotherapy after different reconstruction mode operations (**Table 4**). For N0 patients, receiving PMRT after autologous reconstruction was associated with worse BCSS (AHR, 1.841; 95% CI, 1.055-3.214; p = 0.032). Nevertheless, radiotherapy did not affect the prognosis of patients in N1 and N2 stages, regardless of which reconstruction mode they received. For N3 patients, both autologous reconstruction and combined reconstruction could improve OS and BCSS after radiotherapy, but the prognosis of patients with implant reconstruction was not affected by radiotherapy.

**Table 4:** Adjusted hazard ratio for OS and BCSS associated with radiotherapy after different reconstruction methods in patients with different AJCC_N stages.

Age, y	Autologous		Implant		Combined	
		AHR (95% CI)	P	AHR (95% CI)	P	AHR (95% CI)	P
N0						
OS	1.524 (0.885-2.623)	.128	1.154 (0.672-1.982)	.604	1.813 (0.741-4.433)	.192
BCSS	1.841 (1.055-3.214)	.032	1.515 (0.857-2.678)	.153	2.193 (0.872-5.511)	.095
N1						
OS	0.922 (0.655-1.298)	.640	1.002 (0.726-1.383)	.988	0.633 (0.323-1.239)	.182
BCSS	1.023 (0.709-1.477)	.902	1.008 (0.718-1.415)	.963	0.587 (0.280-1.232)	.159
N2						
OS	0.784 (0.488-1.259)	.313	0.902 (0.512-1.591)	.722	1.417 (0.525-3.826)	.492
BCSS	0.887 (0.543-1.451)	.633	0.981 (0.537-1.792)	.950	1.686 (0.561-5.066)	.352
N3						
OS	0.481 (0.277-0.837)	.010	0.866 (0.495-1.513)	.613	0.233 (0.077-0.704)	.010
BCSS	0.527 (0.297-0.934)	.028	0.840 (0.471-1.495)	.552	0.271 (0.088-0.834)	.023

### Nomograms

**Figure 8:**
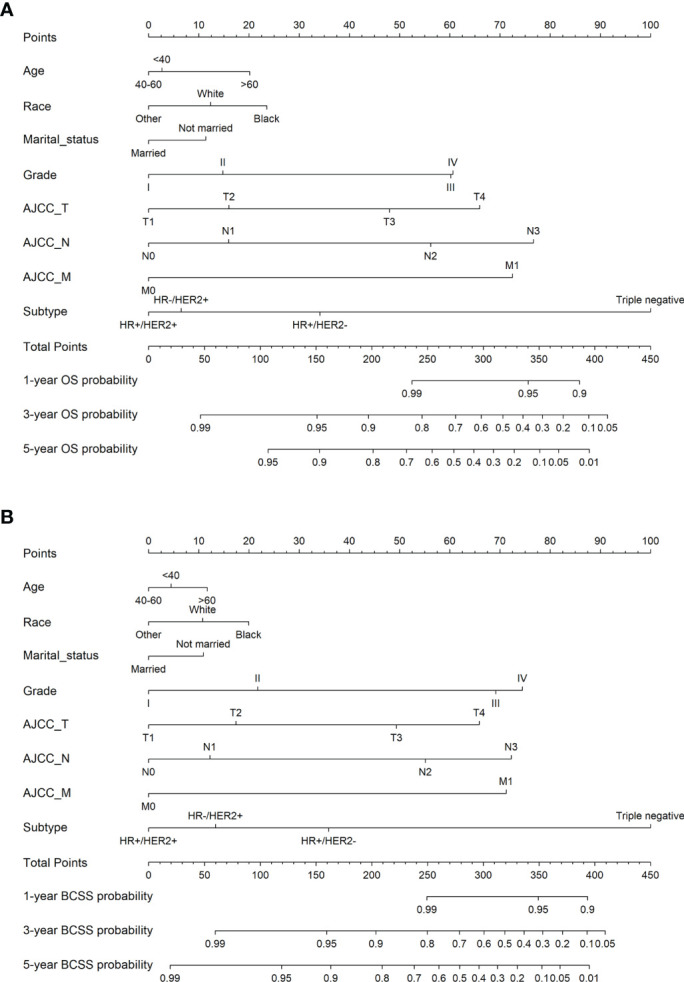
1-, 3-, 5-year probability prediction of overall survival **(A)** and breast cancer specific survival **(B)** in radiotherapy patients. OS, overall survival; BCSS, breast cancer-specific survival.

**Figure 9:**
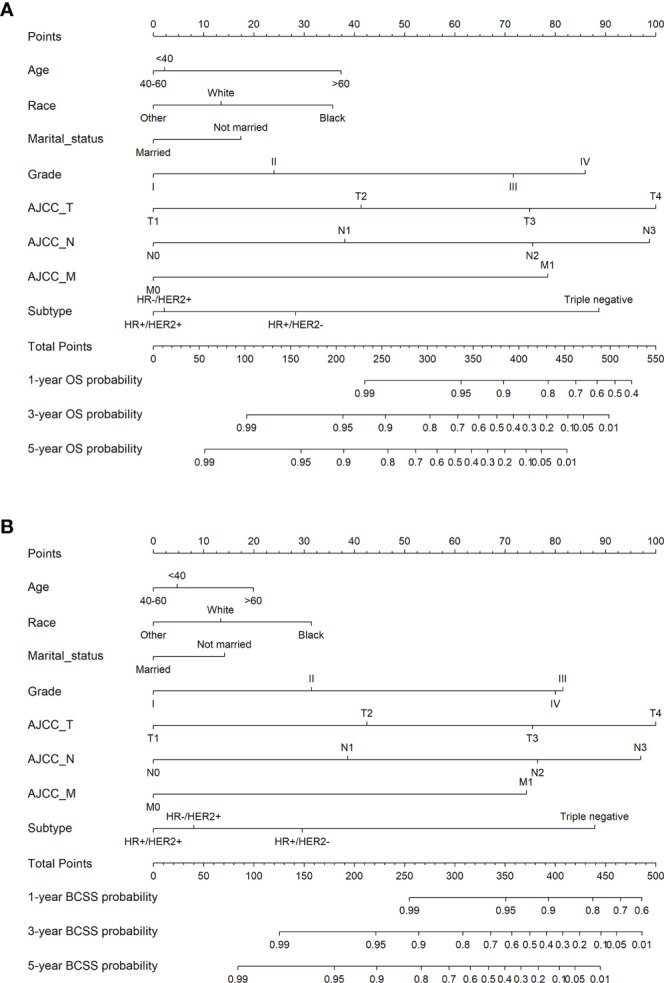
1-, 3-, 5-year probability prediction of overall survival **(A)** and breast cancer specific survival **(B)** in non-radiotherapy patients. OS, overall survival; BCSS, breast cancer-specific survival.

**Figure 10:**
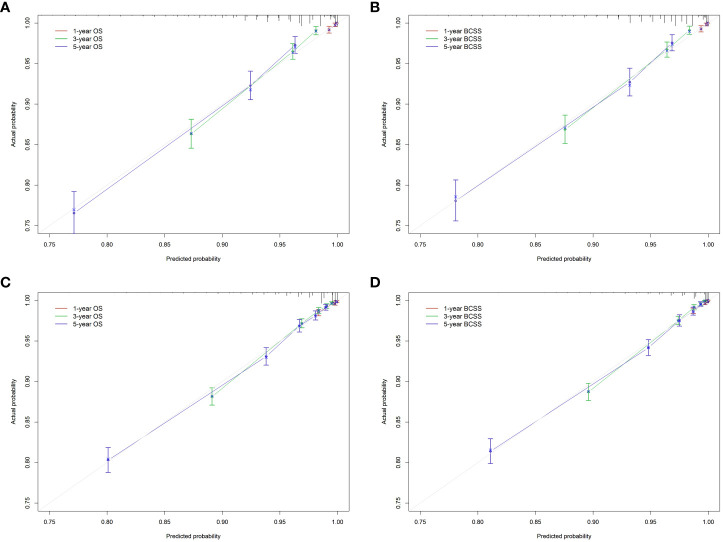
Calibration curve of the nomograms. **(A)**, OS in radiotherapy group. **(B)**, BCSS in radiotherapy group. **(C)**, OS in non-radiotherapy group. **(D)**, BCSS in non-radiotherapy group. OS, overall survival; BCSS, breast cancer-specific survival.

We developed nomograms to predict the prognosis of patients receiving and not receiving PMRT, respectively (**Figures 8** and **9**). Based on the results of multivariate Cox regression, variables such as age, race, marital status, grade, TNM stage, and breast cancer subtype were included in the nomograms. Each prognostic factor corresponded to a specific score, and the sum of each value was compared with the linear predictor to obtain the probability prediction of OS and BCSS at 1-, 3-, and 5- year. The C-indexes of OS and BCSS nomograms predicted by radiotherapy patients were 0.778 and 0.786, respectively, while those in the non-radiotherapy group were 0.818 and 0.847. As shown in **Figure 10**, the calibration curves also reflected the accuracy of the survival prediction model.

## Discussion

In this study, we focused on breast cancer patients who received IBR, to assess the effectiveness of radiotherapy for their prognosis. By using the National Cancer Institute’s SEER database, we can obtain the data based on the entire U.S. population. Previous studies have used this database to compare the prognosis of different reconstruction methods, and some studies have analyzed the prognosis of patients with PMRT. But few studies have evaluated the difference in prognosis based on patients receiving radiotherapy after breast reconstruction technology.

Through demographic data statistics, we found that from 2010 to 2015, 24.9% (5529) of the patients who underwent immediate reconstruction after mastectomy (22218) received PMRT, and those who did not receive PMRT accounted for 75.1% (16689). The number of patients receiving radiotherapy increased slightly in fluctuations. This may be a manifestation of the relaxation of radiotherapy indications, or it may represent the increasing comfort of radiotherapy in the context of breast reconstruction. Secondly, patients with BC after reconstructive treatment have longer life expectancy, so they inevitably face the risk of NBCSD. Therefore, we established a competing risk model to exclude the impact of other causes of death on survival analysis. The cumulative incidence risk curves showed that the risk of BCSD was always higher than that of NBCSD. As for BCSD, the long-term risk of radiotherapy group was higher and higher than that of non-radiotherapy group. Despite all this, we cannot draw the conclusion that radiotherapy is unfavorable to the prognosis of patients. After all, from the radiotherapy guidelines, patients receiving PMRT are already at high risk. Therefore, we stratified the patients according to the lymph node metastasis, and then used Kaplan-Meier survival curves and log-rank test to evaluate the impact of radiotherapy on the prognosis of patients. We found that compared with patients without radiotherapy, patients with low N stage showed a weak survival disadvantage after radiotherapy, while patients with high N stage showed survival benefit after radiotherapy. Due to the imbalance of the baseline distribution of patients in phase N0 (**Table 1**), the number of people without radiotherapy was more than ten times that of people receiving radiotherapy, so we cannot directly conclude that radiotherapy was not conducive to the prognosis of patients with N0. Based on clinical practice, this only showed that radiotherapy had no obvious effect on patients with stage N0. Moreover, the prognosis of patients with stage N3 was poor due to the presence of the high-risk factors, but they showed survival benefits after radiotherapy. This reflected the therapeutic value of PMRT in patients with high stage N. Interestingly, radiotherapy was an adverse factor in univariate Cox regression, but the difference was not statistically significant in multivariate Cox analysis. Similarly, in univariate analysis, the risk of implant reconstruction and combined reconstruction was lower than that of autologous reconstruction, and the histological type was the highest risk of ductal cancer. Nevertheless, the risk ratios of different reconstruction methods and histological types were not significantly different (P > 0.05). Age, race, marital status, grade, TNM stage and subtypes of breast cancer were the key factors affecting survival in both univariate and multivariate Cox regression. We also stratified the patients according to the lymph node metastasis, and calculated the AHRs of radiotherapy after different reconstruction operations. An important finding was that radiotherapy after autologous reconstruction or combined reconstruction can improve OS and BCSS in N3 patients. Finally, this study constructed nomograms for survival prediction, which can effectively predict OS and BCSS after radiotherapy or non-radiotherapy for BC patients. The calibration curves exhibited that the model had good discrimination (C index between 0.778 and 0.847).

The compatibility of PMRT with IBR has been the focus of debate in the field of BC. Radiation oncologists and plastic surgeons both have reservations about the use of IBR under the need of PMRT. PMRT always seems to be associated with reconstruction related complications, such as removal of prosthesis or tissue expander in implant reconstruction and fat necrosis in autologous reconstruction (20–25, 33, 34). Nevertheless, a single-center retrospective cohort study (35) showed that radiotherapy after autologous reconstruction had no negative impact on aesthetic outcomes, and did not increase postoperative complications. A meta-analysis (36) found that when PMRT was delivered after breast reconstruction, morbidity of autologous reconstruction is less than that of implant reconstruction. Specifically, the latter one is more likely to face reconstruction failure, surgical site infection, and eventual repeat surgery (37, 38). Hsin-hua Lee et al. (39) showed that for breast cancer patients requiring PMRT, immediate autologous reconstruction did not affect long-term clinical outcomes. Another study underlined that the type of reconstruction did not affect the late toxicity rate. Radiotherapy after IBR showed acceptable late toxicity and had no effect on OS (40). The literature has various views on the effect of radiotherapy in the setting of IBR. Similarly, in the case of possible PMRT, there is no consensus on the best management and timing of breast reconstruction.

Most of the known studies support that radiotherapy after reconstruction does not affect the long-term survival of BC patients. Our study also found that for stage N3 patients, radiotherapy after autologous reconstruction was associated with improved prognosis. Although we found that radiotherapy after autologous reconstruction was associated with poor BCSS in patients with stage N0, this conclusion should be treated with caution. According to the latest radiotherapy guidelines for breast cancer (20), radiotherapy is not recommended for patients with stage N0, unless combined with high-risk factors. Therefore, we cannot affirm the impact of radiotherapy on the prognosis of patients with stage N0 in the presence of their own high-risk factors. Randomized controlled prospective experimental research is also needed to guide clinical practice.

To accurately identify patients who can benefit from radiotherapy and help make personal suggestions, this study constructed survival prediction nomograms. Although some nomograms have been developed to predict the individual survival probability of BC patients (41, 42), there are still some unique characteristics in our model. First, based on specific radiotherapy disputes, patients who experienced IBR were accurately included in the study participants. Secondly, to exclude the possible bias of the results, we included as many prognostic factors as possible according to the clinical significance. Finally, in addition to the OS rate, the BCSS rate was also reported to predict the patients’ survival probability, thereby avoiding the effect of additional confounding factors associated with the patient’s health.

This study also has several limitations. The first inevitable flaw is the inherent bias in any retrospective study. Second, although some radiotherapy complications can also interfere with the prognosis of patients, due to the lack of this information in SEER database, we cannot consider the impact of complications on the long-term survival rate of patients at the same time. Third, we only compared the radiotherapy group and non-radiotherapy group, and did not distinguish the specific effects of different radiotherapy schemes on the prognosis of patients. The current radiotherapy guidance information in the setting of IBR is lacking (43).

## Conclusions

Our study demonstrates that radiotherapy can improve OS and BCSS in N3 stage breast cancer patients undergoing immediate autologous reconstruction after mastectomy. The survival prediction model constructed in this study can help clinicians quantify the benefits of PMRT after IBR, so as to make personalized treatment recommendations and decisions. Accurate prediction of PMRT can avoid radiotherapy related complications, reduce the incidence of unplanned surgery, and improve the prognosis and survival rate of patients.

## Data availability statement

Publicly available datasets were analyzed in this study. This data can be found here: https://seer.cancer.gov.

## Author contributions

LD and HC designed the study. YB, LH and SL extracted and analyzed the data. XZand ZZ interpreted the evidence and wrote the manuscript. HW, HK and MX revised the article. All authors contributed to the article and approved the submitted version.

## Funding

The National Natural Science Foundation of China (No.82103129); Basic Research Program of Natural Science Foundation of Shaanxi Province (No.2021JQ-422), Prior Science and Technology Program for Overseas Chinese Talents of Shaanxi Province (No.2020-015), and Key Research and Development Program of Shaanxi Province (No. 2022KW-01).

## Acknowledgments

We acknowledge the data support of the SEER program, as well as the Instrument Analysis Center of Xi’an Jiaotong University.

## Conflict of interest

The authors declare that the research was conducted in the absence of any commercial or financial relationships that could be construed as a potential conflict of interest.

## Publisher’s note

All claims expressed in this article are solely those of the authors and do not necessarily represent those of their affiliated organizations, or those of the publisher, the editors and the reviewers. Any product that may be evaluated in this article, or claim that may be made by its manufacturer, is not guaranteed or endorsed by the publisher.
